# Polyphenol Profiling by LC QTOF/ESI-MS and Biological Activity of Purple Passion Fruit Epicarp Extract

**DOI:** 10.3390/molecules28186711

**Published:** 2023-09-20

**Authors:** Monika Siniawska, Aneta Wojdyło

**Affiliations:** Department of Fruit, Vegetable and Nutraceutical Plant Technology, Wrocław University of Environmental and Life Sciences, 37 Chełmońskiego Street, 51-630 Wrocław, Poland

**Keywords:** *Passiflora edulis* Sims, passion epicarp fruits extract, chromatography, in vitro enzyme inhibition effect

## Abstract

A polyphenolic preparation in the form of the passion fruit epicarp extract was analyzed to identify and quantify the polyphenolic compounds using LC QTOF/ESI-MS and UPLC-PDA-FL. The analyzed parameters included antidiabetic activity (α-amylase, α-glucosidase, and pancreatic lipase), inhibitory activity toward cholinesterase (AChE, BuChE), anti-inflammatory activity (COX-1, COX-2, 15-LOX) and antioxidant activity based on ORAC and ABTS. The polyphenolic preparation of the passion fruit epicarp extract contained 51 polyphenolic compounds representing five groups—flavones (25 compounds; 52% of total polyphenolic), flavonols (8; 16%), flavan-3-ols (6; 7%), phenolic acids (4; 3%), and anthocyanins (7; 21%), with derivatives of luteolin (13 derivatives) and apigenin (8 derivatives) as dominant compounds. The preparation was characterized by an antioxidant activity of 160.7 (ORAC) and 1004.4 mmol Trolox/100 mL (ABTS^+o^). The inhibitory activity toward α-amylase, α-glucosidase, and pancreatic lipase reached IC_50_ of 7.99, 12.80, and 0.42, respectively. The inhibition of cholinesterases (IC_50_) was 18.29 for AChE and 14.22 for BuChE. Anti-inflammatory activity as IC_50_ was 6.0 for COX-1, 0.9 for COX-2, and 4.9 for 15-LOX. Food enriched with passion fruit epicarp extract has a potentially therapeutic effect.

## 1. Introduction

Passion fruit (*Passiflora edulis* Sims) is morphologically the largest and the most popular fruit in the botanic family of Passifloraceae. It originates from Brazil but is now cultivated in the tropical and sub-tropical regions of Asia, Africa, America, and Australia [1,2]. It is also one of the “super-fruits,” which are rich in biologically active compounds, including polyphenolic compounds, vitamin C, and carotenoids. The main polyphenolic compounds in passion fruit are anthocyanins, phenolic acids (especially chlorogenic and ferulic acids), as well as flavones and flavonols, specifically derivatives of luteolin, apigenin, and quercetin. These compounds show strong antioxidant properties, neutralize the activity of free radicals, and reduce mutagenic and cancerogenic effects [2,3]. Moreover, passion fruit has many health benefits; for example, it exhibits antiviral, antifungal, antibacterial, as well as tranquilizing, and anxiolytic therapeutic properties [1]. Owing to its high content of biologically active compounds, regular intake of passion fruit helps to treat non-communicable diseases caused by improper lifestyle, including stress, malnutrition, and environmental conditions.

The mesocarp has a sweet taste with an astringent note; therefore, passion fruit can be consumed both fresh and processed. Thanks to its attractive sensory profile, passion fruit is also a popular raw material for juice making, or it is combined with other foodstuffs [1,4]. The mesocarp constitutes 50% of the passion fruit; therefore, its processing leaves a significant amount of waste in the form of epicarp, mesocarp, endocarp, and seeds [1], which are rarely made use of. The disposal of waste products is becoming more and more of a problem among manufacturers because it is an expensive process in which a lot of energy and other utilities are used. Therefore, alternative solutions are sought for the specific waste material generated in the food processing industry [5,6]. Attempts have been made to reuse pomace from, for instance, pineapple, mango, avocado, sugar apple, papaya, guava, and lychee [1]. It has been demonstrated in many cases [7,8,9] that pomace has an attractive chemical composition, which encourages its reuse and indicates various industries for the purpose, e.g., the pharmaceutical, food-processing, and cosmetic industries. It appears that pomace still has a high content of desirable nutrients, such as minerals, protein, or fiber; therefore, it is used as an additive in the production of fruit tea, bakery products, confectionery, and alcoholic beverages [6,10,11]. The current trends in the use of pomace involve the manufacturing of edible dyes to be added in the process of biofilm making or food packaging or for making new-generation cosmetics [12,13,14,15]. It has also been noted in many cases [16,17] that pomace is useful as a material for isolating biologically active compounds, including phenolic, tetraterpenoid, triterpenoid compounds, and many others. Therefore, it is desirable to determine not only the nutrient content of pomace but also that of bioactive compounds. Such information will help decide on its potential uses in various industries, e.g., pharmaceutical, cosmetic, or food processing industry, for instance, in product fortification to improve the content of bioactive compounds and the health-beneficial value of newly developed products. Despite the high potential of waste product utilization, it is not always possible to use directly all types of waste products as additives to foodstuffs or in other industries. It has become a trend recently to isolate valuable, biologically active components from waste products, for instance, polyphenolic compounds, which demonstrate a wide array of health-beneficial properties [5].

The few earlier literature reports [18] confirm the high bioactive potential of passion fruit peel and seeds. Several research studies on the use of passion fruit mesocarp (peel) focused on the use of its by-products as a raw material characterized by a considerable content of desirable dietary fiber [19,20]. The addition of fiber-rich ground mesocarp to extruded breakfast cereals lowered their glycemic index by about 50% in a model in vitro study vs. the glycemic index of white bread, as shown by Leoro et al. [21]. Kandandapani et al. [22] used extracts of passion fruits mesocarp and seeds to evaluate the antidiabetic potential in diabetic rats following streptozotocin (STZ)-induced oxidative stress. However, in their study, the authors did not provide a detailed composition of the bioactive fraction and only stated that the extract contained steroids, flavonoids, and triterpenoids as major secondary metabolites [22]. Macagnan et al. [23] reported on the use of passion fruit peel as an alternative source of dietary fiber. They evaluated the effect of a diet enriched with apple pomace, orange bagasse, and passion fruit peel on the health of rats [23]. A diet with the addition of passion fruit mesocarp waste extract helped to maintain a normal blood glucose level, resulting in lower postprandial hyperinsulinemia. It also prevented dysfunction or dysregulation of pancreatic β cells and, as a consequence, protected the rats against insulin resistance, obesity, and atherosclerosis [23]. Farid et al. [24] examined the impact of the oral intake of purple passion fruit peel extract on alleviating pain and stiffness and improving the physical activity of adults with gonarthrosis. A preliminary study has shown that the typical symptoms accompanying gonarthrosis were reduced in such adults after the administration of the purple passion fruit peel extract [24].

All these reports clearly indicate that passion fruit and its various parts are an attractive waste material for reuse, offering a wealth of potential applications. Although a research study using an animal model, as well as other studies, were conducted, there has been no report on the complete characterization of the polyphenolic fraction of the purple passion fruit epicarp extract.

Therefore, the aims of this research study were to (*i*) obtain a polyphenolic extract of the passion fruit epicarp, (*ii*) perform its qualitative (QTOF/ESI-MS) and quantitative analysis (UPLC-PDA-FL) to determine its polyphenolic compound content, and (*iii*) assess the biological potential of the isolated fractions relating to the antioxidant, antidiabetic, anticholinesterase, and anti-inflammatory activity of the passion fruit epicarp extract (ePFe), which was rich in polyphenolic compounds.

The quantitative and qualitative examination of the polyphenolic profile of passion fruit epicarp extract and the assessment of its biological potential will enable the fortification of foodstuffs with programmable health-beneficial qualities.

## 2. Results and Discussion

### 2.1. Content of Polyphenolic Compounds in Purple Passion Fruits Epicarp Extracts—Identification and Quantification

Polyphenolic compounds in the isolated polyphenolic fraction of the passion fruit epicarp extract were analyzed using liquid chromatography coupled with electrospray ionization-quadrupole-time of flight-mass spectrometry (LC-QTOF/ESI-MS), both in the negative (phenolic acid, flavone, flavonols, flavan-3-ols) and positive (anthocyanins) ionization modes, depending on the analyzed group of compounds. The compounds in the analyzed extract were identified based on available reference standards, standard data provided in the published literature, retention times, and mass fragmentation. Flavonoids usually show two main absorption bands. Band I is within 320–385 nm, and band II is within 250–285 nm, and they represent ring absorption for the structure of molecules B and A, respectively. Flavonols and flavones were identified as glycosides having one or more sugar groups. Moreover, the obtained identification results indicate that the number and position of the sugars and the hydroxyl groups, and the presence of aromatic acids and aliphatic acids connected with the sugars, determine the formation of many derivatives of flavonoids and phenolic acids. These respective data are shown in Table 1.

In this study, the structural identification of polyphenolic compounds carried out with QTOF/ESI-MS enabled the identification of 51 polyphenolic compounds representing five groups—flavones (**25** compounds), flavonols (**9** compounds), flavan-3-ols (**6** compounds), phenolic acids (**4** compounds), and anthocyanins (**7** compounds) (Table 1). Many of those compounds are identical to those previously reported in the literature for *Passiflora* species de Souza et al., Es-Safi et al., Simirgiotis et al. and Zucolotto et al. [25,26,27,28], confirming that the various compounds were correctly identified [29,30,31,32,33].

**Figure 1:**
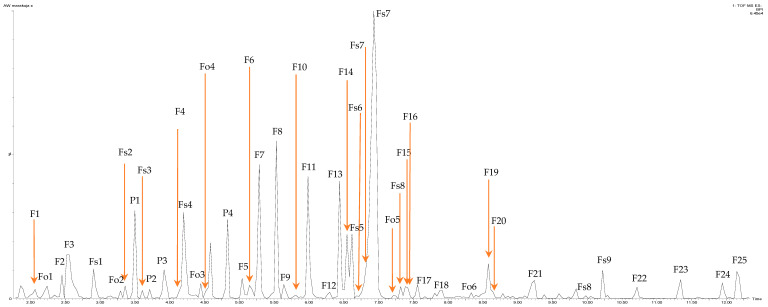
Chromatogram LC-MS/QTOF of the epicarp passion fruits extract analyzed in negative ESI mode: flavan-3-ols (Fo1-Fo6), phenolic acid (P1-P4), flavone (F1-F25) and flavonols (Fs1-Fs9). The designations indicate the specific polyphenolic compound, for an explanation, see Table 1.

The corresponding total ion chromatogram (TIC) is shown in Figure 1. Additionally, the determination of procyanidin polymers was carried out, and the degree of polymerization (DP) was computed. The total identified polyphenolic compounds in the polyphenolic fraction of the passion fruit epicarp extract (ePFe), found after purification of the material to remove ballast substances, was 14126.73 mg/100 g d.m. The dominant components of the preparation were flavones (52%) >> anthocyanins (21%) > flavonols (16%) > flavan-3-ols (7%), and finally, phenolic acids comprising 3% of the total content of polyphenols

The obtained polyphenolic preparation of passion fruit epicarp extract contained 26 flavone derivatives, including both *C*-glycosylated and *O*-glycosylated flavones, derivatives of luteolin, apigenin, acacetin, and chrysoeriol. These two types of bonds are easily identified based on MS/MS fragmentation patterns. The bonds in *C*-glycosylated flavones feature a characteristic substitution of saccharides directly attached to aglycone in ring A through the C-C bond, and all of them have substituents in positions 6 (C-6) and/or 8 (C-8) and/or 7 (C-7) of the aglycone group. The C-C bond in the *C*-glycosylated flavonoids is resistant to breaking; therefore, the cleavage mainly occurs at sugar bonds at the side of *O*-glycosylated flavonoids. In addition, the sugar residue at C-6 shows greater fragmentation than at C-8 or C-7. The *C*-glycoside conformation of quercetin derivatives rarely occurs in nature.

The dominant flavones in the analyzed ePFe were luteolin (*m*/*z* 285; **13** compounds) and apigenin (*m*/*z* 269; **7** compounds), accounting for 38% and 34%, respectively, of all the flavones. The content of all apigenin derivatives was 2419.22 mg/100 g and was slightly lower than that of luteolin (2727.53 mg/100 g). The content of acacetin (*m*/*z* 283) and chrysoeriol (*m*/*z* 299) was 14% each (Table 1). In the analyzed ePFe, the flavone of the highest concentration (968.45 mg/100 g) was a compound identified as a chrysoeriol derivative. Although luteolin derivatives had the largest representation (**13** compounds), the highest concentration of luteolin in any of those compounds was 430 mg/100 g (Table 1).

Luteolin, apigenin, and their derivatives are important compounds often found in fruit and vegetables, both as glycosides and aglycone [34]. According to earlier reports, luteolin and its *C*-glycosylated derivatives have antioxidant properties, and they also play a major role in the inhibition of oxidative stress, which may lead to many diseases, including diabetes mellitus (DM), inflammatory conditions, Alzheimer’s disease (AD), and Parkinson disease (PD) [34]. Recent literature sources demonstrate that apigenin has certain unique and desirable biological properties; for instance, it is responsible for the biological protection of internal body organs (heart, brain, liver, and lungs), biological hypotension, has a hypoglycemic and hypolipemic effect, antioxidant, and anti-inflammatory properties, and it prevents osteoporosis and improves immune regulation [35]. In addition, the consumption of apigenin, in contrast with other flavonoid compounds, namely myricetin or kaempferol, may reduce the risk of ovarian cancer [36]. Therefore, it should be noted that the presence and availability of flavone derivatives for consumption are beneficial for human health. The passion fruit epicarp extract is an interesting source of these compounds, providing potential pharmacological or pharmaceutical effects upon introduction into the human diet.

Flavonols are the best visible flavonoids in fruits and vegetables, and quercetin is the most frequently consumed flavonol. Flavonols were the second major group of polyphenols we identified in the epicarp of passion fruit. In ePFe, we identified nine derivatives of quercetin (*m*/*z* 301), mainly non-acylated flavonoid glycosides (seven compounds) associated with a sugar residue, such as a pentoside and a hexoside, or a rutinoside. Quercetin-3-di-sinapoyl-tri-glucoside-7-di-glucoside was at the highest concentration, whereas the lowest was that of quercetin of -3-*O*-pentoside, -3-*O*-hexoside, and -3-*O*-acetylglucoside (less than 100 mg/100 g; Table 1). In addition, quercetin and its derivatives are very common in plants, and they show significant and multifaceted biological activity. They are known to be therapeutic in inflammatory conditions, cardiovascular disease, atherosclerosis, cancers, arthritis, or allergic reactions. The compounds also have a neuroprotective pharmacological effect, preventing cerebral neurodegenerative disorder, AD, PD, and many other conditions. Therefore, the presence and availability of quercetin for consumption are very important for the human body to help it maintain proper homeostasis.

In the case of anthocyanins, we detected their typical representatives, that is, cyanidin (*m*/*z* 287) and delphinidin (*m*/*z* 303) derivatives (Table 1). A total of seven anthocyanins were identified, including non-acylated 3-hexoside and -pentoside and their corresponding acylated or *p*-coumaroyl derivatives. Cyanidin derivatives dominated over delphinidin derivatives, as they accounted for 70% of the compounds.

Anthocyanins are natural, water-soluble pigments present in some parts of fruit, vegetables, flowers, and seeds, in which they also modulate their sensory properties (color). Similar to other flavonoids, anthocyanins show their biological activity and therapeutic potential mainly through the mechanisms underlying their antioxidant and anti-inflammatory properties because oxidative stress and inflammation are common factors triggering many human diseases, including non-communicable diseases, for instance, lifestyle ones. Literature data indicate that anthocyanins have neuroprotective, cardioprotective, and antidiabetic properties; they protect against obesity, cancer, and retinopathy [37]. Based on a meta-analytical approach, Fallah et al. [38] described the effect of dietary anthocyanins in the form of a powder/extract, either pure or enriched with anthocyanins, on the biomarkers of oxidative stress and antioxidant properties in humans. The study results demonstrated that anthocyanins observably reduced the level of oxidative stress markers, including cholesterol, low-density lipoproteins (LDL), ox-LDL, and peroxidation of lipids (MDA and isoprostanes) when consumed in amounts ranging from 1.7 to 1230 mg/day for 2 to 26 weeks. They also considerably improved antioxidant capacity, as expressed by TAC and SOD levels. The recognizable therapeutic activity of anthocyanins was confirmed in vitro, in vivo, and epidemiological studies. The results indicate that anthocyanins are characterized by a highly promising pharmaceutical activity if taken regularly.

In the preparation of the passion fruit epicarp extracts, we identified procyanidin monomers (*m*/*z* 289) and dimers (*m*/*z* 577), which were quantitatively determined as polymeric procyanidins by the phloroglucinol analysis method (Table 1). The content of the monomers as (+/−)-(epi)-catechin and polymeric procyanidins of ePFe was 1017.05 mg/100 g. The degree of polymerization was 7.43 (Table 1).

A diet rich in apple, cocoa, herbal plants, blueberry, green tea, or grapes still provides the essential dietary sources of flavan-3-ols, yet new sources of these compounds are sought [39]. Flavan-3-ols in food account for such qualitative parameters of food as astringency, bitterness, acidity, sweetness, fragrance, and color, as well as the viscosity of saliva. The higher the degree of polymerization of a given raw material or the products made of it, the more perceptible such sensory properties of the food as astringency and bitterness, that contribute to its reduced consumer acceptability. The daily intake of flavan-3-ols may vary between 10 mg and 0.5 g/person/day, and the compounds most probably consumed are the B1 and B2 dimers [39]. Moreover, flavan-3-ols also improve the microbiological, oxidative, and thermal stability of food [39].

The pharmacological and biological properties of flavan-3-ols have been thoroughly investigated to reveal their anti-inflammatory, anticancer, and antiviral activities and their role in the protection of the cardiovascular system [39]. Especially desirable are their antioxidant properties, which are based on various mechanisms, for example, chelation of transient metals (iron, copper, aluminum), scavenging of free radicals (peroxyl and nitric oxide, hydroxyl, alkoxyl, superoxide anion), and non-radicals (singlet oxygen, ozone, peroxynitrite, hydrogen peroxide, hypochlorous acid).

The sample of passion fruit epicarp extracts was found to contain four derivatives of polyphenolic acids, but their concentrations were low. The test sample also contained sugar derivatives of ferulic, sinapic, and salicylic acids. The total amount of these compounds was 409.14 mg/100 g (Table 1). Salicylic acid was dominant, and the other acids were present at lower relative concentrations. Phenolic acids, though their number was the smallest among all the polyphenolic compounds in ePFe, are usually omnipresent and have well-documented health-protective effects, basically as antimutagenic, anti-inflammatory, antimicrobial, anticancer, and antidiabetic agents [40].

Literature data show no detailed compositional analysis with identification of polyphenolic fraction components in passion fruit epicarp has been reported before. According to Fonseca et al. [41], a detailed analysis of polyphenolic composition was performed for juices, seeds, and pulp. Reis et al. [4] analyzed the composition of the pulp, peel, and seeds of yellow, purple, and orange passion fruit, but not the epicarp, and determined the fractions and the selected bioactive components as the total content of phenolic compounds quercetin, and kaempferol. More details were reported for the content of anthocyanins, whereby delphinidin- and malvidin-3,5-*O*-glucoside, cyanidin, and pelargonidin-3-*O*-glucoside and their aglycone were identified. The total identified anthocyanins amounted to 103.69 mg/100 g of d.m., and kaempferol derivatives were present in the amount of 74.70 mg/100 g of d.m. [4].

Another study by KidØy et al. [42] identified anthocyanin compounds, such as cyanidin-3-*O*-glucoside (97%) and small amounts of cyanidin-3-*O*-6-malonyl-glucoside (2%) and pelargonidin-3-*O*-glucoside (1%) in the peel of passion fruit. Reis et al. [4] reported that the sum of the polyphenolic compounds, identified using the Folin–Ciocalteu method in the passion fruit mesocarp, was 1570.80 mg/100 g of d.m. A review article by Fonseca et al. [41] described the presence of different luteolin and quercetin derivatives, such as basic flavones and flavonols, and additionally some chrysin and apigenin in the peel and seeds. Phenolic acids, as derivatives of hydroxycinnamic and hydroxybenzoic acids (e.g., salicylic acid, ferulic acid, and others), had a large representation in the analyzed seeds. It should be emphasized that the compounds identified in the juice or seeds [41] corresponded, to some extent, to those detected in the epicarp in the present study.

According to a review by Fonseca et al. [41], the peel and pulp of purple passion fruits were found to contain flavonols such as kaempferol, kaempferol-3-*O*-glucoside, quercetin-3-*O*-glucoside, quercetin-3,7-di-*O*-hexoside, quercetin-7-*O*-glucoside in the following amounts: 0.74, 0.33, 1.67, 0.58, and 0.33 mg/g of d.m., respectively; flavones, such as luteolin, luteolin-6-*C*-glucoside and luteolin-8-*C*-(2-*O*-rhamnosyl)-hexoside in the amounts of 20.00, 41.00, and 0.44 mg/g of d. m., respectively, as well as anthocyanins (in μg per g of d.m.): cyanidin -3,5-*O*-diglucoside (14.8 μg), cyanidin (12.4 μg), cyanidin-3-*O*-glucoside (5.78 μg), delphinidin (0.91 μg), delphinidin-3,5-*O*-glucoside (86.8 μg), and pelargonidin-3-*O*-glucoside (15.5 μg). The other identified polyphenolic compounds were 5-hydroxy-3,3′,4′,6,7-pentamethoxyflavone (artemitin), quercetin, quercetin-glucoside and -3-*O*-(6″-acetyl-glucoside), -3-*O*-(6″-malonyl-glucoside)-7-*O*-glucoside, luteolin-glucoside and luteolin-3-glucosyl-rhamnoside, -8-C-digitoxoside, -8-C-neohesperoside, -8-C-β-boivinopyranoside, -8-C-β-digitoxopyranoside, -8-C-β-digitoxopyranosyl-4′-*O*-β-D-glucopyranoside, -rhamnosyl-glucoside, chrysin-8-C-(2″-*O*-α-6-deoxy-glucopyranosyl)-β-D-glucopyranoside, cyanidin-glucoside, cyanidin-3-(6″-malonylglucoside), phloretin-glucoside, quercetin-3-*O*-(6″-acetyl)-glucosyl-2″-sinapic acid, protocatechualdehyde acid and protocatechuic acid, and (+/−)-(epi)-catechin, (−)-epicatechin-glucoside isomer. Many of these, as well as other compounds, were first detected in the analyzed passion fruit epicarp extracts, as presented in Table 1.

### 2.2. In Vitro Biological Activity of Passion Fruit Epicarp Extract

Polyphenols are built of numerous phenol units and hydroxyl groups, which gives them certain physical, chemical, and, first of all, biological properties. Biological activity is a term that can be interpreted in many ways. It should also be examined by various methods to verify health benefits, especially in the case of substances with the desirable composition of bioactive compounds. Therefore, in this study, the biological activity of ePFe is presented as the antidiabetic, anticholinesterase, anti-inflammatory, and antioxidant capacity. All the results are shown in Table 2.

#### 2.2.1. Antidiabetic Activity

Diabetes is a chronic endocrine disease that disturbs the correct metabolism and normal body functioning. It is characterized by sustained high blood sugar levels. Its treatment is based, among other things, on the reduction in postprandial hyperglycemia by slowing down glucose decomposition and absorption by inhibition of enzymes, such as α-amylase and α-glucosidase, that hydrolyze carbohydrates. Plant-based products rich in biologically active compounds, such as phenolic compounds, coumarins, and terpenoids, have for years been believed to be capable of reducing blood glucose levels [43,44,45].

The mixture of ePFe compounds demonstrated inhibition of enzymatic activity, which was observed for α-amylase, α-glucosidase, and pancreatic lipase in the amounts corresponding to the respective IC_50_ values of 7.99, 12.8, and 0.42 (Table 2). Previously, in vivo studies on animal models indicated that the antidiabetic activity associated with flavonoids outcome from their modulating effects on sugar, starch, and cellulose metabolism, improvement of reduction in insulin action and resistance, and pancreatic β-cell function, inflammation, and oxidative stress in muscles, and reduction in triglyceride levels and cholesterol synthesis. Antidiabetic activity is associated with the molecular mechanisms of phenolic compounds in pathways of AMP-activated protein kinase, peroxisome proliferator-activated receptor, glucose transporter, hepatic enzymes, NF κB, β-cell apoptosis, and tyrosine kinase inhibitor. Additionally, their activity is associated with hydroxyl groups and α, β ketones [22,25,45].

Sebastian et al. [44] reported that passion fruit considerably inhibited α-amylase with IC_50_ of 146.04 μg/mL. Another study by Kandandapani et al. [22] confirmed the antidiabetic effect of the passion fruit peel. STZ-induced diabetes in rats was considerably reduced after the administration of extracts from dried peels and seeds of passion fruit. The antidiabetic effect of the extracts from dried peels and seeds of passion fruit was observed within 72 h from STZ administration and was maintained till the end of the experiment. The blood glucose level in the control rats with diabetes was reduced, whereas the blood glucose level in the diabetic rats treated with the extract from the dried peel and seeds of passion fruit was ideal, ranging from 6.00–8.00 mmol/L through the entire time of the study, that is, for 14 days after administration of the first dose.

Polyphenolic fractions derived from plants have for many years been believed to help combat diabetes. Inhibition of α-amylase was observed for polymeric procyanidins obtained from raspberry, green tea, strawberry, grapes, and cocoa trees. Proanthocyanidins (tannins) and ellagic acid derivatives from batatas leaves demonstrated observable inhibition of α-amylase [43]. Inhibition of α-glucosidase was observed in the case of quinic acid and quercetin-3-*O*-glucosidase, whereas quercetin was found to inhibit pancreatic lipase [46]. Phenolic acids also have a beneficial effect on the activity of glucose and insulin receptors. They increase the expression of the glucose transporter GLUT2 in pancreatic β cells (which produce insulin) and promote the translocation of GLUT4 along the protein kinase pathways. The same mechanism of transporter stimulation is observed for ferulic acid, which is an effective antidiabetic drug [40]. Among all the properties of phenolic acids, the best effects were observed for the inhibition of the two essential enzymes in DM, namely, α-glucosidase and α-amylase, which are responsible for the transformation of dietary carbohydrates into glucose [40].

Pancreatic lipase is the most important lipolytic enzyme in the gastrointestinal tract. It is responsible for the hydrolysis of triglycerides into monoglycerides and fatty acids, enabling the uptake of fatty fractions and nutrients. Inhibition of pancreatic lipase plays an important role in the prevention and treatment of obesity since it significantly reduces the digestion of fats, thus limiting their accumulation in adipocytes [46].

#### 2.2.2. Inhibition of Acetylcholinesterase (AChE) and Butyrylcholinesterase (BuChE)

The progressing aging of populations has recently aroused interest in identifying natural inhibitors of AChE and BuChE for use as therapeutic agents in neurological disorders, such as Alzheimer’s disease or Parkinson’s disease [46,47].

The passion fruit epicarp extract we analyzed in this study was capable of inhibiting AChE and BuChE with IC_50_ of 18.29 and 14.22, respectively (Table 2). Such results should be perceived as showing a significant potential in using ePFe for the inhibition of AChE and BuChE. There have been no earlier reports documenting the use of the passion fruit epicarp extract for this purpose. Enzymes of AChE and BuChE, despite many structural similarities, presented some differences in activity. It is known that the hydroxyl and methoxy groups in bioactive compounds can enhance the enzyme inhibitory effect due to stronger binding capacity mainly as hydrogen bond, π-π, and hydrophobic interactions.However, the issue of docking polyphenols with acetylcholinesterase enzymes is still poorly studied and explicated, and these differences are seen in the degree of similarity of the inhibitors to enzyme molecules [39,48,49,50,51].

Lim et al. [48] examined AChE and BuChE inhibition by several cultivars of kiwi fruit. The results ranged from 19.46 to 39.25% at 0.833 mg/mL for AChE and from 18.51 to 53.20% at 0.833 mg/mL for BuChE inhibition, depending on the cultivar. Lim et al. (2014) mentioned that AChE inhibition capacity most significantly correlated with total phenolics and vitamin C content. BuChE inhibition did not significantly correlate with total phenolics, but a significant correlation with total flavonoids was noted. The results showed that the content of total phenolics and vitamin C in kiwi had a desirable effect on AchE inhibition. On the other hand, it is worth noting that vitamin C may not be effective in AChE inhibition, as shown in AChE inhibition tests, where vitamin C inhibited less than 10% AChE when used at a concentration of 0.42 mM (the results were not published).

In their study, Jabir et al. [49] cited numerous literature sources that confirm a strong AChE and BuChE inhibition by quercetin. The flavonol quercetin is found in many fruits and vegetables, such as onion, apple, potato, broccoli, and grape. In a study by Khan et al. [52] on the AChE and BuChE inhibition by quercetin, the reported IC_50_ values were 353.86 and 420.76 μmol/L for AChE and BuChE, respectively. According to Esmaeili et al. [53], AChE inhibition by quercetin isolated from *Agrimoniapilosa ledeb* achieved IC_50_ at 19.8 μmol/L, and the compound was proposed as a potential therapeutic agent for the treatment of Alzheimer’s disease.

Luteolin is an important bioflavonoid found in vegetables and flowers in two forms—aglycone and glycosides [52]. Choi et al. [34] investigated, among other things, the anti-Alzheimer activities of luteolin and its *C*-glycosylated derivatives. Luteolin showed the lowest AChE and BuChE inhibitory activity with IC_50_ of 9.27 and 9.60 μM. Orientin and isoorientin were characterized by a definitely stronger AChE and BuChE inhibitory activity with IC_50_ of 20.06, 11.05 μM for orientin, and IC_50_ of 29.48 and 11.33 μM for isoorientin.

Sezen Karaoğlan et al. [53] investigated the AChE and BuChE inhibitory activity of apigenin-7-glucoside and luteolin-7-glucoside. The latter showed a much stronger inhibitory activity, amounting to 65% for AChE and 36% for BuChE. Regarding apigenin-7-glucoside, the results were 30% for AChE and 13% for BuChE.

In a study by Mira et al. [54], quercetin showed the strongest AChE inhibitory activity of all the compounds found in *Angelica shikokiana* Makino: IC_50_ at 35.5 μM. Luteolin showed inhibitory activity toward AChE with an IC_50_ above 500 μM.

The results published by Wu et al. [55] demonstrated that the activities of myricetin, quercetin, fisetin, and gallic acid were the highest of all active components of Phyllanthus emblica Linn. because of their low docking results and a strong AChE inhibitory activity: IC_50_ at 0.1974, 0.2589, 1.0905, and 1.503 mM, respectively. Moreover, myricetin, quercetin, and fisetin reversibly inhibited AChE.

#### 2.2.3. Anti-Inflammatory Activity

Cyclooxygenase (COX-1, COX-2) produces prostaglandins involved in the inflammatory conditions accompanying disorders and diseases such as diabetes, cancer, or atherosclerosis [42]. COX-1 is expressed in the gastric mucosa and endothelial cells and, most probably, has the function of a “housekeeping enzyme,” maintaining their integrity and contributing to the normal functioning of the cardiovascular system by releasing prostacyclin (PGI2). COX-2 is induced by many types of inflammation factors and plays an important role in the biosynthesis of prostaglandins associated with the inflammatory response [56]. Recently, researchers have focused on the health benefits of secondary metabolites in treating inflammatory conditions, but it is still important to search for new phenolic-rich plant sources [57,58,59]. Lipoxygenase (LOX) is present in living plants and animals. Its role is to catalyze the oxidation of polyunsaturated fatty acids (PUFA) to hydrogen peroxides of fatty acids [59]. Earlier studies provided evidence explaining the connection between 15-LOX and the growth and development of neoplastic cells, whereas 15-LOX inhibitors also show anti-inflammatory activity [58,59,60]. LOX and COX function as stimulants in inflammatory conditions such as cancer, asthma, osteoporosis, allergy, atherosclerosis, and joint inflammation [58].

The mixture of compounds in the ePFe was characterized by a potential inhibitory activity toward COX-1, COX-2, and 15-LOX, expressed as IC_50_ of 6.00, 0.90, and 4.90, respectively (Table 2). It is worth noting that there have been no previous reports on the inhibitory activity of passion fruit peel extract toward COX-1, COX-2, or 15-LOX. Polyphenols regulate and control pro-inflammatory cytokines’ synthesis resistance by interfering with immune cell regulation and can suppress toll-like receptor (TLR) and pro-inflammatory genes’ expression. Additionally, they inactivate NF-κB, inhibit phosphatidylinositide 3-kinases/protein κB, and modulate nitogen-activated protein kinase and arachidonic acids pathways. They inhibit certain enzymes involved in reactive oxygen species production, i.e., xanthine oxidase and NADPH oxidase (NOX), while they upregulate other endogenous antioxidant enzymes such as peroxidase (Px), catalase, superoxide dismutase (SOD), and glutathione (GSH). In addition, polyphenolic compounds inhibit COX, LOX, and phospholipase A2, leading to a reduction in the production of prostaglandins and leukotrienes and inflammation antagonism [25,29,57,58,59].

According to Albuquerque et al. [59], the passion fruit by-product water extracts do not show any ability to reduce the level of nitric oxide produced by lipopolysaccharide-stimulated macrophages because there was no significant difference between the level of nitric oxide after treatment with the passion fruit by-product water extracts and the lipopolysaccharide-stimulated control cells.

#### 2.2.4. Antioxidant Capacity

Free-radical scavenging ability is a very important aspect in the evaluation of biological activity because free radicals contribute to many disorders and diseases, such as cancer, degenerative, cardiovascular, and neurological diseases, as well as diseases of the lungs [60].

The passion fruit epicarp extract was characterized by a free-radical scavenging ability of 1004.4 mmol TE/100 g and 160.7 mmol TE/100 g, as measured by ABTS and ORAC assays (Table 2).

Reis et al. [4] reported that purple passion fruit peel scavenged free radicals with IC_50_ of 9.37  ±  0.05 g/100 mL, measured by ABTS. According to Suleria et al. [61], passion fruit peel was characterized by ABTS free-radical scavenging ability of 1.04 ± 0.07 mg ascorbic acid equivalents (AAE)/g. Passion fruit examined by Septembre-Malaterre et al. [62] showed the highest antioxidant ability among all the fruit types examined, including banana, lychee, mango, papaya, and pineapple, which reached 14.08 ± 1.99 μM TE, as measured by ORAC.

Moo-Huchin et al. [63] measured the free-radical scavenging ability by ABTS and DPPH method for the freeze-dried peel of purple star apple, yellow cashew, and red cashew. The antioxidant capacity was lower than that of the extract, and the difference was significant.

Flavonoids demonstrate free-radical scavenging ability because they have a catechol group on the B ring, a double bond between C2 and C3, and an oxygen atom at the C4 position. The role of the catechol group is to donate hydrogen/electrons to stabilize the radical. The function of the double bond is to bind transient metal ions such as iron and copper. Luteolin is a flavonoid with a high antioxidant potential because it has two typical features of flavonoids on which their free-radical scavenging ability is based [52,53].

Flavan-3-ols exhibit antioxidant properties by means of several mechanisms, including free-radical scavenging ability, metal chelation, as well as enzyme inhibition, and mediation. Their electron configuration enables an easy transfer of electrons to and from free-radical species, and this way, the radical nature of reactive oxygen forms is transferred onto flavan-3-ols. They are regarded as stronger antioxidants than vitamins C and E. Flavan-3-ols, and their microbiological metabolites of phenolic acids are particularly good antioxidants because a derivative radical is more stable and less harmful than the initial radical species. According to literature reports, flavan-3-ols have a better antioxidant ability than flavonols because the oxidation of flavan-3-ols gives mainly semiquinone radicals, the coupling of which produces mainly oligomeric compounds by nucleophilic addition. Such coupling maintains the number of reactive catechol/pyrogallol structures, and the free-radical scavenging ability is thus preserved. On the other hand, flavonols form quinones, which are more liable to undergo the redox cycle and potentially act as prooxidants [39,61,62].

Phenolic acids show antioxidant properties because of the reactivity of their phenolic molecule—the hydroxyl group, connected with the aromatic ring. The main mechanism of its action is free-radical scavenging by donating a hydrogen atom from the reactive phenolic molecule [35].

Tian et al. [64] compared, among other things, the antioxidant ability of luteolin and quercetin by DPPH, ABTS, and FRAP methods. The antioxidant ability of luteolin was higher than that of quercetin in each case. Luteolin was characterized by antioxidant capacity, expressed as IC_50_ of 2.09 μg/mL and 0.59 μg/mL for DPPH and ABTS, respectively, and 0.04 mmol Fe^2+^/μg/mL for FRAP. On the other hand, quercetin was characterized by IC_50_ of 1.84 μg/mL and 0.51 μg/mL for DPPH and ABTS, respectively, and 0.04 mmol Fe^2+^/μg/mL for FRAP.

## 3. Materials and Methods

### 3.1. Materials

The study material of passion fruit epicarp was used. A homogeneous powder was obtained after freeze–drying (Alpha 1-4 LSC, Christ, Germany) extraction with water:ethanol (50:50, *v*/*v*) at a ratio of 3:1 (solvent:powder). The extraction was repeated 5 times after a 1 h maceration with mixing every 15 min, and all of the solvent was collected and mixed after a 10 min centrifugation at 15,000× *g* (MPW-380R; Warsaw, Poland). After that, all of the ethanol was evaporated (RV 10, IKA; Königswinter, Germany). To isolate polyphenolic compounds, water extract was absorbed into a column packed with Amberlite XAD-16 (Sigma-Aldrich, Darmstadt, Germany), according to the procedure described by Wojdyło et al. [65]. The mixture of polyphenols was then eluted out using 80% ethanol, evaporated again, freeze-dried, and finally used as a powder for all analyses (Section 2.2.2, Section 2.2.3 and Section 2.2.4).

### 3.2. Identification and Quantification of Phenolic Compounds of Passion Fruit Epicarp Extract 

The qualitative (LC-QTOF/ESI-MS) and quantitative (UPLC-PDA-FL) analyses of polyphenols (flavan-3-ols at 280 nm, flavones and flavonols at 360 nm, phenolic acids at 320 nm, and anthocyanins at 520 nm) were conducted as described earlier by Wojdyło et al. [66] and Nowicka et al. [67]. Individual polyphenols were separated using the LC-QTOF/ESI-MS and ACQUITY UPLC BEH C18 column (1.7 µm, 2.1 × 100 mm; Waters Corporation, Milford, USA) operated at 30 °C. The analysis was performed at the following conditions: sample injection volume 5 µL, elution time 15 min, gradient and isocratic sequence, flow rate 0.42 mL/min. The program was commenced at a gradient elution of 99 to 65% using solvent A (0–12 min), after which the percentage of solvent A was reduced to 0% for the column conditioning (12.5–13.5 min), and then the gradient was restored to the initial conditions (99% A) at 15 min. The mobile phase consisted of solvent A (2% and 0.1% formic acid in H_2_O (*v*/*v*) for LC-QTOF/ESI-MS and UPLC-PDA, respectively analysis) and solvent B (100% acetonitrile). All measurements were repeated three times. The results were expressed in mg/100 g of dry matter (d.m.). The calibration curves were made for the standard luteolin-7-*O*-glucoside (*y* = 11,211*x* + 498,134; *r^2^* = 0.9835), apigenin-7-*O*-glucoside (*y* = 20,768 + 121,323*x*; *r^2^* = 0.9912) at concentrations ranging from 0.05 to 0.5 mg/mL.

Two MS experiments were performed, one in negative and positive ionization before and after fragmentation. Characterization of the single components was carried out via the retention time and the accurate molecular masses. Analyses were carried out with voltage ramping cycles from 0.3 to 2 V, using full scan mode, and collision-induced fragmentation experiments were performed using argon as the collision gas. The optimum values of LC-MS parameters were capillary and cone voltages of 2500 V and 30 V, respectively. The capillary temperature was set to 300 °C, while the source heater temperature was 100 °C, drying gas (nitrogen) flow rate of 300 L/h. Leucine enkephalin had a flow rate of 2 µL/min and was used as the reference compound at a concentration of 500 pg/µL, and *m*/*z* at 554.2615 and 556.2771 were detected for negative and positive ionization, respectively. Data processing was performed using MassLynx 4.0 ChromaLynx Application Manager software (https://www.waters.com/waters/en_US/MassLynx-Mass-Spectrometry-Software-/nav.htm?cid=513164&locale=en_US accessed on 1 August 2023).

### 3.3. Analysis of Polymeric Procyanidins by Phloroglucinolysis

The polymeric forms of procyanidins were analyzed using the method described earlier by Wojdyło et al. [65].

The analysis was carried out in the UPLC-FL apparatus. Phloroglucinolysis products were separated on a BEH Shield C18 RP column (1.7 µm, 2.1 × 100 mm; Waters Corporation, Milford, USA) with solvent A (2.5% acetic acid in H_2_O, *v*/*v*) and solvent B (100% acetonitrile). The cycle was set with the following gradient: 0 min 3% B, 0.5–5 min up to 9% B, 5.1–15 min up to 16% B, and 15–45 min up to 50% B, followed by the column rinsing and conditioning. The analysis was conducted at the column temperature of 30 °C, flow rate of 0.42 mL/min, and sample injection volume of 5 µL. Fluorescence was recorded at an excitation wavelength of 278 nm and an emission wavelength of 360 nm. Calibration curves for the quantitative determination were obtained for procyanidin B2, (+)-catechin and (−)-epicatechin. The average degree of polymerization was calculated as the molar ratio of all flavan-3-ol units to terminal units of (−)-epicatechin and (+)-catechin. All the samples were analyzed in triplicate, and the results were expressed in mg per 100 g d.m. 

### 3.4. In Vitro Biological Activity of Passion Fruit Epicarp Extract

The samples were prepared as described earlier by Wojdyło et al. [65]. A weighed sample of ePFe for in vitro analyses was dissolved in 80% MeOH, subjected to ultrasound treatment for 20 min (Sonic 6D; Polsonic, Warsaw, Poland), and centrifuged (MPW-55; Warsaw, Poland) at 19,000× *g* at 4 °C for 10 min to obtain a clear extract for use in the analyses.

#### 3.4.1. Inhibition of α-Amylase, α-Glucosidase, and Pancreatic Lipase

The analysis was carried out as described earlier by Wojdyło et al. [65,66,68].

Inhibitory activity toward α-amylase and α-glucosidase was measured as a result of a reaction and incubation at 37 °C. Absorbance was measured at 600 nm for α-amylase and 405 nm for α-glucosidase. Standard samples contained a buffer instead of the enzymes, and acarbose was used for the control.

Pancreatin lipase inhibition was measured based on the amount of *p*-nitrophenol formed from *p*-nitrophenyl acetate. The samples were incubated at 37 °C and absorbance was measured at 400 nm. Standard samples contained a buffer instead of the enzymes, and orlistat was used for the control.

The results of inhibitory activity toward α-amylase, α-glucosidase, and pancreatic lipase were expressed as IC_50_ (mg/mL of extracts).

#### 3.4.2. Inhibition of Acetylcholinesterase (AChE) and Butyrylcholinesterase (BuChE)

The analysis was carried out using the method described earlier by Wojdyło et al. [65,68]. The substrate in the form of acetylcholine iodide and butyrylcholine chloride was hydrolyzed by the enzyme to obtain thiocholine which, in a reaction with 5,5′-dithiobis-(2-nitrobenzoic acid) produced colorful products of the inhibition in the form of 2-nitrobenzoate-5-mercaptothiocholine and 5-thio-2-nitrobenzoate, detected at 405 nm. Standard samples contained a buffer instead of the enzymes, and galantamine was used for the control.

The results were expressed as IC_50_ (mg/mL) inhibitory activity toward AChE and BuChE.

#### 3.4.3. Anti-Inflammatory Activity

The analysis was carried out using the method described earlier by Wojdyło et al. [65]. Anti-inflammatory activity was expressed as cyclooxygenase (COX-1 and COX-2) inhibition by enzymes and was assessed using the method described earlier in the protocol COX Inhibitor Screening Assay Kit (Cayman, No. 560131). Inhibitory activity toward 15-lipoxygenase (15-LOX) was measured using the method of ferrous oxidation-xylenol orange (FOX) published by Turkiewicz et al. [60].

The results for COX-1, COX-2, and 15-LOX inhibition were expressed as IC_50_ (mg/mL).

#### 3.4.4. Antioxidant Capacity

Antioxidant activity was measured based on ABTS and ORAC assays. The analysis was carried out using the method described earlier by Wojdyło et al. [65,66] and Nowicka et. al. [67]. ABTS was measured at 734 nm after running the reaction for exactly 6 min. In the case of ORAC, the potential of passion fruit epicarp extract was measured in 5 min intervals at the excitation and emission wavelength of 493 nm and 515 nm, respectively, for 50 min.

The results were expressed as mmol Trolox equivalent (TE)/100 g.

## 4. Conclusions

The isolation of polyphenolic compounds is desirable as it provides products having a high potential, measured *in vitro* as biological activity. The passion fruit epicarp extract has a high content of polyphenolic compounds, mainly flavones (represented by **26** compounds), flavonols (**8** compounds), anthocyanins (**7** compounds), and flavan-3-ols. It should be noted that the utilized material is a new product with new potential in application. This provides a sustainable alternative source of such compounds, compatible with the closed-loop bioeconomy. Otherwise, the further use or disposal of the material would pose a considerable environmental problem.

The methods that enable the isolation of bioactive compounds as a mixture of polyphenolic compounds can be used in many industries. Therefore, the passion fruit epicarp extract in future may be used as an admixture in the production of functional foods (i.e., food for the fortification of solid or liquid/semi-liquid products, such as jellies, juices, smoothies, jams, etc.), also as integral dietary supplements, because of its therapeutic effects, specifically, its anti-inflammatory properties, blood glucose lowering effect, or its ability to prevent cell damage resulting from free-radical oxidation.

Additionally, it would be beneficial to conduct future additional analyses on the passion fruit epicarp extract, i.e., with cell culture methods or animal studies, to gain a deeper understanding of its biological activity and the underlying mechanisms of its potential health-promoting effects. Cell culture experiments can explain the extract’s impact on specific cell lines, providing insights into cellular processes, gene expression, and signaling pathways. Animal studies, on the other hand, offer a broader perspective on knowing the extract’s effects on living organisms and can help validate its potential health benefits.

## Figures and Tables

**Table 1:** Characterization of individual phenolic compounds of the endocarp passion fruits extracts using their spectral characteristic of the compounds using LC-QTOF/ESI-MS in negative (flavan-3-ols, phenolic acid, flavone, flavonols) and positive (anthocyanins) ionization mode and their quantification [mg/100 g].

Peak No	t_R_ (min)	Assigned Identity	Molecular Ion [M-H]^−^ (*m*/*z*)	Main Ions MS/MS (*m*/*z*)	Phenolic Content [mg/100 g dw]
Flavan-3-ol					
Fo1	2.30	(+)-catechin ^†^	289.0143	245.0367	35.02 ± 1.43
Fo2	4.40	(−)-epicatechin ^†^	289.0143	245.0363	38.43 ± 2.65
Fo3	3.30	Procyanidin dimer (B2) ^†^	577.0538	289.0143	943.60 ± 35.27 ^¥^
Fo4	4.50	Procyanidin dimer	577.0311	289.0152	
Fo5	7.28	Procyanidin dimer	577.0237	289.0153	
Fo6	8.20	Procyanidin dimer	577.0538	289.0143	
				*Σ flavan-3-ols*	1017.05
				*Degree of polymerization*	7.03 ± 0.23
Phenolic acid					
P1	3.50	Ferulic acid 4-*O*-glucoside	355.0124	313.0664/269.0807/**193.0318**/161.0304	51.95 ± 2.43
P2	3.71	Sinapic acid hexoside	385.0844	190.0087	121.53 ± 1.45
P3	3.92	Salicylic acid glucoside	299.0522	**137.0487**	152.12 ± 2.76
P4	4.83	Ferulic acid 4-*O*-galactoside ^†^	355.0754	269.0807	83.54 ± 2.99
				*Σ phenolic acid*	409.14
Flavone					
F1	2.21	Acacetin-rhamnoside	429.0596	283.0466	499.34 ± 10.32
F2	2.45	Apigenin derivatives	455.1432	305.0297/287.0388/**269.0135**/168.9985	75.73 ± 2.43
F3	2.52	Acacetin-rhamnoside	429.05	**283.0432**/207.0354/151.0095/165.0272	305.34 ± 6.54
F4	4.15	Luteolin-8-*C*-glucoside (orientin) ^†^	447.0599	417.0670/**285.0168**/151.9977	223.54 ± 11.32
F5	5.04	Apigenin-7-*O*-(6″-galloyl)-hexoside	583.1554	357.1140/327.0977/**269.0874**/165.0430	273.23 ± 15.21
F6	5.18	Luteolin-7-*O*-(6″-galloyl)-rhamnoside	583.1653	537.1574/357.1180/281.1142/285.0874	288.34 ± 16.32
F7	5.29	Apigenin-6-*C*-(6″-*O*-crotonyl)-pentoside	469.1075	361.1308/340.0760/294.0700/269.0807/161.0304	360.23 ± 10.32
F8	5.53	Di-hydroxy-di-methoxy-flavone-di-hexose (chrysoerior derivatives)	683.2097	**486.1270**/440.1263/307.0784/**300.9991**	968.45 ± 21.27
F9	5.64	Apigenin-6-*C*-xyloside-6-*C*-glucoside (vicenin)	563.1574	537.1557/341.0632/269.0269/203.0748/165.0351	55.43 ± 1.32
F10	5.81	Apigenin-6-*C*-xyloside-6-*C*-glucoside (vicenin)	563.1557	424.0364/327.0903/**269.0269**	192.32 ± 2.74
F11	5.99	Luteolin-7-*O*-rutinoside ^†^	593.1002	**285.0168**	199.48 ± 5.29
F12	6.30	Luteolin-8-*C*-digitoxopyranosyl-4′-*O*-hexoside	577.1099	285.0168	104.23 ± 3.22
F13	6.44	Luteolin-*O*-di-pentoside-*O*-hexoside	711.1668	355.0754/311.0916/**285.0168**	460.56 ± 5.25
F14	6.55	Apigenin-2″-*O*-deoxyhexosyl-*C*-hexoside	577.1148	413.0587/336.0821/**269.0807**	505.29 ± 2.76
F15	7.34	Luteolin-7-*O*-rutinoside ^†^	593.1102	**285.0098**/284.0084	178.44 ± 5.11
F16	7.38	Luteolin-*O*-deoxyhexosyl-*C*-pentoside	561.1247	523.1760/320.1076/359.1804/**285.0168**/277.0204/195.0452	66.85 ± 2.88
F17	7.56	Luteolin-8-*C*-glucoside (orientin) ^†^	447.0555	285.0168	114.69 ± 10.32
F18	7.91	Luteolin-6-hydroxy-*O*-glucuronide	477.0694	359.1804/303.1126/**285.0168**	94.38 ± 3.54
F19	8.16	Luteolin-8-*C*-digitoxopyranosyl-4′-*O*-hexoside	577.1198	285.0098	93.26 ± 2.55
F20	8.58	Acacetin-di-rhamnoside	575.1001	411.0390/301.0133/**283.0432**	193.48 ± 6.44
F21	9.21	Luteolin-8-*C*-digitoxo-pyranosyl-4′-*O*-hexoside	577.1148	415.0667/353.0414/311.0339/**285.0202**	337.48 ± 8.45
F22	10.71	Luteolin-8-*C*-digitoxo-pyranosyl	415.0709	311.0339/**285.0202**	135.32 ± 1.34
F23	11.34	Luteolin derivatives	697.3668	859.4095/285.9161	430.96 ± 4.21
F24	11.94	Apigenin-*C*-hexosyl-*O*-rhamnoside-*O*-hexoside	741.3559	533.3114/399.2210/271.0691/**269.0202**	401.00 ± 11.54
F25	12.15	Apigenin-6-*C*-(6″-*O*-crotonyl)-glucoside	499.2872	533.2972/**269.0202**/171.0862/127.0954	555.99 ± 24.12
				*Σ Acacetin dervatives*	998.16
				*Σ Apigenin derivatives*	2419.22
				*Σ Luteolin derivatives*	2727.53
				*Σ Chrysoerior derivatives*	968.45
				*Σ flavone*	7341.47
Flavonols					
Fs1	2.91	Quercetin-3-*O*-pentoside	433.0339	380.1268/313.0664/241.0921/146.0696	89.93 ± 3.56
Fs2	3.36	Quercetin-3-*O*-hexoside (glucoside I) ^†^	463.0523	301.0062	59.45 ± 5.32
Fs3	3.60	Quercetin-3-*O*-hexoside	463.0523	**301.0062**/385.0443/137.0100	64.32 ± 2.14
Fs4	4.20	Quercetin-3-*O*-hexoside	465.0722	301.0666	228.11 ± 6.89
Fs5	6.62	Quercetin-3-*O*-rutinoside ^†^	609.1018	301.0133	219.45 ± 6.99
Fs6	6.78	Quercetin-3-*O*-galactoside ^†^	463.0523	301.0054/276.0603/245.07	140.41 ± 5.27
Fs7	6.93	Quercetin-3-di-sinapoyl-tri-glucoside-7-di-glucoside	761.1526	336.0821/301.0054/276.0671/230.0474	1504.15 ± 2.56
Fs8	7.32	Quercetin-3-*O*-acetylglucoside	505.0633	**301.0054**	89.79 ± 3.76
Fs9	10.22	Quercetin-*O*-tri-pentoside-3-*O*-hexoside	859.4095	694.0944/301.1803/271.0724	156.76 ± 5.21
				*Σ flavonols*	2324.11
Anthocyanins					
A1	4.59	Cyanidin-3-*O*-glucoside ^†^	449.0603	**287.0180**	737.69 ± 3.11
A2	6.29	Cyanidin-3-*O*-(6″-*p*-coumaroyl)-glucoside	595.1019	449.0646/299.0216/287.0180/249.0563	789.9 ± 5.74
A3	6.55	Delphinidin-3-*O*-(6″-*p*-coumaroyl)-glucoside	611.1003	465.0489/**303.0134**	774.82 ± 6.32
A4	6.72	Delphinidin-3-*O*-glucoside ^†^	465.0577	303.0098	85.72 ± 2.45
A5	6.86	Cyanidin-3-*O*-(6″-*p*-coumaroyl)-glucoside	595.0870	449.0646/287.0146	98.44 ± 4.11
A6	8.48	Cyanidin-3-*O*-hexoside-rhamnoside	595.1069	431.0513/287.0180	500.43 ± 5.72
A7	8.84	Delphinidin-di-*C,C*-hexosyl-*O*-rhamnoside	759.0952	456.0952/**303.0062**	47.96 ± 3.87
				*Σ anthocyanins*	3034.96
				*Total polyphenols*	14 126.73

^†^t_R_, MS and MS/MS, compared with the standard compound; The characteristic ions are marked by bold type; t_R_, retention time; ^¥^ sum of procyanidins dimer as polymeric procyanidins for compounds Fo3, Fo4, Fo5, Fo6. The *m/z* values of the predominant ions are given in bold type.

**Table 2:** In vitro biological activity (ABTS, ORAC, α-amylase, α-glucosidase, AchE, BuChE, COX 1 and 2, LOX-15) of the epicarp passion fruits extract.

Name of Analysis	Biological Activity
Antioxidant activity [mmol TE/100 g]	
-ABTS^o+^	1004.40 ± 20.80
-ORAC	160.70 ± 16.35
Antidiabetic activity [IC_50_; mg/mL]	
-α-amylase	7.99 ± 0.23
-α-glucosidase	12.80 ± 1.01
-pancreatic lipase	0.42 ± 0.02
Cholinesterase activity [IC_50_; mg/mL]	
-acetylocholinesterase	18.29 ± 1.76
-butyrylcholinesterse	14.22 ± 1.11
Anti-inflamatory activity [IC_50_; mg/mL]	
-cyclooxygenase 1	6.00 ± 0.74
-cyclooxygenase 2	0.90 ± 0.63
-15-lipoxygenase	4.90 ± 0.39

## Data Availability

Data is contained within the article.

## Sample Availability

Samples of the compounds passion fruit epicarp extract are available from the authors.
